# Optimization of hemophilia B treatment via population PK modeling of rIX-FP, including a 3-week regimen

**DOI:** 10.3389/fped.2025.1710546

**Published:** 2025-12-15

**Authors:** Naoki Terasaka, William Mckeand

**Affiliations:** 1 Medical Affairs Department, CSL Behring K.K., Kita-Aoyama Minato-ku, Tokyo, Japan; 2 Clinical Pharmacology Department, CSL Behring, King of Prussia, PA, United States

**Keywords:** children and adolescents, dosing regimen, extended half-life, factor IX, Hemophilia B, population pharmacokinetics, prophylaxis, treatment optimization

## Abstract

**Background:**

Recombinant factor IX–albumin fusion protein (rIX-FP) enables extended-interval prophylaxis for hemophilia B. While higher trough levels with reduced dosing frequency have been shown vs. standard FIX products, age-related differences in pharmacokinetics (PK) may constrain extended intervals in children. We aimed to refine a population PK (popPK) model of rIX-FP to inform clinically practical dosing across pediatric, adolescent, and adult patients.

**Methods:**

Pooled FIX activity data from 113 previously treated patients (1–63 years) in five clinical studies were analyzed with a two-compartment popPK model. Covariates included body weight, age, weight-adjusted dose, and ethnicity (Japanese vs. non-Japanese). Model performance was evaluated by standard diagnostics and prediction-corrected visual predictive checks. Simulations estimated single-dose duration above 5% FIX activity and steady-state troughs for common prophylactic regimens stratified by age (<6, 6–<12, 12–<18, ≥12, ≥18 years).

**Results:**

Body weight significantly influenced clearance (CL) and volumes; weight-normalized CL was faster in children, especially <6 years. No meaningful ethnic effect was detected; PK parameters and simulated profiles were comparable between Japanese and non-Japanese patients. Simulations showed that in patients ≥12 years, median steady-state troughs were maintained around ∼5% with 25–50 IU/kg weekly, 50 IU/kg every 10 days, 75 IU/kg every 14 days, and 100 IU/kg every 21 days—supporting a 3-week option in well-controlled patients. In contrast, children <6 years generally required weekly dosing (≥40–50 IU/kg) to achieve ∼5% troughs; extending intervals substantially reduced the proportion meeting target levels.

**Conclusion:**

rIX-FP supports individualized prophylaxis with extended intervals up to 3 weeks in patients ≥12 years while younger children typically require more frequent dosing due to higher weight-normalized clearance. These PK-based insights can guide shared decision-making to balance protection, treatment burden, and patient preference across pediatric and adolescent age groups.

## Introduction

Hemophilia B (HB) is a rare X-linked bleeding disorder caused by a deficiency of coagulation factor IX (FIX) (1). More than 2,100 potential FIX gene mutations have been identified on the long arm of the X chromosome, leading to varying disease severity, ranging from mild (>5% to <40% FIX activity), to moderate (1%–5%), to severe (<1%) (1, 2). Spontaneous or traumatic bleeding into muscles or joints can result in arthropathy, disability, and reduced quality of life (1, 2). Preventing bleeds and maintaining musculoskeletal function remain key goals of prophylaxis (1, 2).

Traditional HB treatment involves regular infusions of plasma-derived FIX or recombinant (rFIX) products, which require frequent administration and may lead to suboptimal outcomes, particularly in severe cases (3–5). Managing bleeding episodes or surgeries often necessitates multiple FIX infusions to maintain adequate coagulation levels. Similarly, prophylactic therapy prophylaxis 2–3 injections per week due to the relatively short half-life of standard FIX products, averaging about 18 h (3). The development of long-acting FIX products, such as recombinant coagulation factor IX-albumin fusion protein (rIX-FP), has significantly advanced hemophilia therapy by extending the half-life, thereby reducing injection frequency, improving convenience and adherence, and lessening the need for repeated venous access, particularly in pediatric patients (6–8).

The efficacy and safety of rIX-FP has also been demonstrated with once every 7 and 14 days prophylaxis in previously treated pediatric as well as adult patients treated with rIX-FP for a mean exposure time of over 12 months (9, 10). The population PK (popPK) model developed previously correlates well with observed clinical data and supports prolonged dosing of rIX-FP with intervals of up to 2 weeks (11). More recently, we conducted a pharmacokinetic model-based assessment comparing rIX-FP with other FIX replacement therapies in severe HB (12).

The current study aimed to provide a more detailed characterization of the popPK of rIX-FP, identify factors contributing to PK variability, and simulate FIX activity levels by incorporating additional data from Study 3003 (13, 14). The popPK model was employed to simulate various rIX-FP dosing scenarios, providing guidance for its use in routine prophylaxis for HB patients. Furthermore, the Japanese popPK model with data from Study 3003 described the FIX activity well in Japanese and non-Japanese patients. Finally, this study data was used in Japan as well as in the EU Partial Change Application (PCA) for adding a new prophylaxis regimen, the Q3W dosing regimen, to the current product information of rIX-FP intravenous (IV).

Study 3003 was designed as an extension of Studies 3001 and 3002. The enrolled subjects were almost entirely rollover participants from those earlier studies, and no new pediatric patients were recruited. While the patient pool remained the same, the newly collected PK data in Study 3003 came from a once-every-three-weeks (Q3W) regimen, which had not been previously evaluated. Therefore, the model's covariate space was not expanded via new subjects, but the pharmacokinetic space was broadened through the inclusion of Q3W data, particularly in pediatric patients. This approach enhanced the previous model by incorporating richer dosing condition data, supporting more robust simulations and regulatory submissions for the extended dosing regimen.

## Methods

### Ethics declaration

The clinical studies included in this analysis were conducted in compliance with Good Clinical Practice (GCP) guidelines and the ethical principles outlined in the Declaration of Helsinki. The protocols were approved by the institutional review boards (IRBs) of all participating sites. All participants provided written informed consent prior to enrollment in the study. Confidentiality of patient data was maintained in accordance with applicable data protection regulations. The data used in this analysis are derived from clinical trials conducted under Good Clinical Practice (GCP) guidelines. Written informed consent was obtained from all individual participants and, in the case of minors, from a parent and/or legal guardian.

### Study population

This population pharmacokinetic (popPK) analysis was conducted using pooled data from five previously conducted clinical studies of rIX-FP (Studies 654_2001, 2004, 3001, 3002, and 3003) (10, 11, 13–17). These studies were designed to evaluate the safety, pharmacokinetics, and efficacy of rIX-FP in previously treated patients with moderate to severe hemophilia B (FIX activity ≤2%). A brief summary of each study is provided below to describe the sources of data used in this model development. The characteristics of the study designs are summarized in Supplementary Table S1.

### Bioanalytical methods

The FIX activities of rFIX-FP were measured using a validated one-stage clotting method (11). Plasma samples of FIX were measured using the Behring Coagulation System (Siemens Healthcare Diagnostics, Marburg, Germany), with a lower limit of quantification (LLQ) of 0.25% of normal and validated ranges of 10%–150% for FIX activity in plasma standard low-dilution (FIX.PSL.d) and 0.25%–15% for FIX activity in plasma standard low-concentration (FIX.PSL.low). Between-run precision, expressed as the relative standard deviation (RSD), was 14.1% for FIX.PSL.d and 9.8% for FIX.PSL.low, while accuracy in FIX-depleted plasma ranged from 91%–114% for FIX.PSL.d and 89%–120% for FIX.PSL.low. FIX activity is expressed as percent of normal (%), which is equivalent to international units per deciliter (IU/dL).

### PK analysis software and methods

PK analysis was performed using NONMEM® Version VII Level 7.4.3 with PDx-Pop Version 5.2.2 as the interface. The First-Order Conditional Estimation Method with Interaction (FOCEI) was used for parameter estimation.

### Population pharmacokinetic model

The analysis used a previously developed popPK model for log-transformed FIX activity based on data from multiple clinical studies (11). Baseline FIX activity was assumed to be ≤2%, consistent with the study inclusion criteria. Observed FIX activity above 2% prior to the first dose was attributed to residual effects of prior FIX products and endogenous FIX activity.

The present analysis updated the dataset to include final previously treated patients (PTP) data from Study CSL654_3003, while retaining the structure of the previously developed population PK model, including the two-compartment disposition, inter-individual variability (IIV), and combined residual error model (additive and proportional).

Observed FIX activity (FTOT) was modeled as the sum of three components: exogenous FIX activity from rIX-FP (FEX), endogenous baseline FIX activity (BASE), and residual FIX activity from prior FIX products (FPP), as follows:

FTOT=FEX+BASE+FPP

FPP was described by an exponential decay function using BSE as the initial value:

FPP=BSE×exp(−[CL/Vc]×TIME)

Here, BSE refers to the observed FIX activity level immediately prior to the first administration of rIX-FP, measured at the time of the last FIX dose administered before study entry. FPP was set to zero when its value declined below the lowest of the following three thresholds: (1) the BSE value, (2) the screening FIX activity level, or (3) 2 IU/dL, which approximates the lower limit of quantification (LLOQ). The same CL and Vc values as those for rIX-FP were applied to model the decay of FPP. This approach was adopted because

(1) the contribution of FPP to total FIX activity was generally small and limited to the initial period after first rIX-FP dosing; (2) available data were insufficient to support independent estimation of FPP-specific CL and Vc; and (3) exploratory modeling with separate parameters yielded unstable estimates without improving model performance.

A small number of predose FIX activity values were reported below the lower limit of quantification (LLOQ; 0.25 IU/dL). These values were retained as observed and included in the model without censoring or imputation. Due to the limited number and minimal deviation below LLOQ, their impact on parameter estimation was considered negligible.

Inter-individual variability (IIV) was evaluated on all key structural pharmacokinetic parameters using a forward inclusion (*p* < 0.01) and backward elimination (*p* < 0.001) approach based on the likelihood ratio test (LRT). IIV was retained in the model only when its inclusion led to a statistically significant improvement in model fit and produced stable parameter estimates with acceptable shrinkage (<30%).

IIV on inter-compartmental clearance (Q) and peripheral volume of distribution (Vp) was tested but excluded from the final model due to lack of statistical significance and unstable estimates with high shrinkage.

Residual error variability was modeled separately for Studies 2001/2004 and Studies 3001–3003. This approach was supported not only by statistical testing but also by differences in study design, sampling schedules, and patient populations, which likely contributed to the higher observed variability in the later studies. Visual inspection of residual plots and descriptive statistics further supported this modeling decision.

### Pharmacostatistical model

The pharmacostatistical model consisted of two components: the inter-individual variability model and the residual error model. The inter-individual variability model described unexplained random variability in individual parameter values, assuming log-normal distribution. Inter-individual variability was estimated for clearance, central volume, and endogenous FIX activity.

Initial model development used a diagonal covariance matrix for inter-individual variability, and attempts were made to define a full block covariance matrix for the final model. If numerical stability issues arose or goodness-of-fit criteria showed no significant differences, diagonal matrices were preferred.

The decision to retain the full block covariance matrix was based on assessments of numerical stability, objective function value (−2 Log Likelihood), and goodness-of-fit diagnostic plots. Only when the full block covariance matrix model was numerically stable and showed clear improvement over the diagonal covariance matrix model in these criteria was it retained; otherwise, the diagonal covariance matrix was preferred.

The residual error model captured variability between observed and predicted values was described using a combined additive and proportional error structure (Supplementary Figure S1).

### Covariate modeling

Body weight, age, BMI, observed predose FIX activity, aspartate aminotransferase (AST) levels, alanine aminotransferase (ALT) levels and creatinine clearance were evaluated as continuous covariates for their potential effects on CL, Vc, and BASE. Body weight-adjusted dose was also evaluated as a continuous covariate on Vc, in addition to body weight, to explore potential distribution effects not fully captured by total dose or weight alone. Although it is mathematically dependent on body weight and dose, it was retained based on statistical significance and its ability to improve model fit without introducing collinearity. Ethnicity, including Japanese and non-Japanese groups, was assessed as a categorical covariate, although individual evaluation of non-Japanese ethnicities was limited by sample sizes. The multi-ethnic nature of the overall dataset supports the robustness of the model across populations. Similarly, categorical covariates such as hepatitis positivity were assessed.

The covariate modeling utilized a full model with a backward deletion approach. Covariates were evaluated by removing them individually from the semi-full model. Statistical significance was assessed at *p* < 0.001, corresponding to an objective function value (OFV) increase of more than 10.83 for a chi-squared distribution with one degree of freedom, applicable to a single-parameter test such as a continuous covariate or a binary categorical covariate. For categorical covariates with more than two levels, the corresponding chi-squared cutoff value was adjusted according to the increased degrees of freedom. Backward deletion continued until all remaining covariates in the model were statistically significant, and those with non-significant effects were not retained in the final model. Continuous covariates were included in the model using linear relationships on log-transformed parameters. Prior to inclusion, pairwise correlation coefficients were assessed to evaluate the potential for collinearity. No substantial collinearity was detected among the tested covariates (|*r*| < 0.5), and covariate effects were retained based on statistical significance and clinical plausibility.

For the effect of Japanese ethnicity, despite the limited number of Japanese PTPs (*n* = 10), this was tested on key PK parameters such as clearance and volume of distribution during the model development process using a likelihood ratio test with *p* < 0.01 for inclusion and *p* < 0.001 for backward deletion. The population PK model included data from multiple ethnicities in global studies. The effect of Japanese ethnicity was specifically tested due to regulatory requirements in Japan, and no significant effect was found despite the small sample size (*n* = 10). Other ethnicities were not tested individually because of limited sample sizes and because the overall model reflects multi-ethnic data, supporting its robustness across populations.

### Model evaluation

To assess the performance of the final popPK model, a prediction-corrected visual predictive check (pcVPC) (18) was conducted (Supplementary Figure S2). This graphical tool evaluates whether model simulations align with the observed data. Observed data were overlaid with the median as well as the 5th and 95th percentiles of concentration–time profiles obtained from 1,000 simulations.

### Simulations

The final popPK model for FIX activity was used to simulate FIX activity profiles under various IV dosing scenarios for different age groups (patients <6 years, 6–12 years, 12–18 years, **≧**12 years, and ≧18 years). These scenarios included single doses (25, 35, 40, 50, 75, and 100 IU/kg) and steady-state doses (25, 35, 40, 50, 75, 100, and 150 IU/kg) administered every 7, 10, 14, 21, or 28 days. Simulations used individual estimated endogenous FIX activity levels and body weights from the patients in the PK model, with a 10-minute infusion duration assumed. A total of 1,000 replicates per scenario were performed, and FIX activities over time were calculated for each simulation.

## Results

### Clinical dataset and patient demographics

In the updated popPK analysis, a total of 113 unique patients (from Studies 2001, 2004, 3001, 3002, and 3003), aged with 1–63 years, contributed 3,387 FIX activity data points, which were used in the popPK analysis of FIX activity data. Those were patients who received rIX-FP treatment and contributed ≥ 1 measurable PK concentration. Of the 113 patients, 12 were under 6 years old, 15 were between 6 and <12 years, 5 were between 12 and <18 years, and 81 were ≥18 years old.

Table 1 presents the basic distributions of covariates by studies where multiple rIX-FP doses were administered. The median observed pre-dose FIX activity was 0.7, 1.6, 1.9, 1.6 and 16.3 IU/dL for Studies 2001, 2004, 3001, 3002 and 3003, respectively. Predose FIX activity levels were higher in Study 3003 for patients who transitioned from Studies 3001 or 3002, as the time from their last FIX dose in Studies 3001 or 3002 to the predose sample in Study 3003 ranged from only 1–16 days, which was insufficient to allow FIX activity levels to return to their true baseline values.

**Table 1:** Summary of patient characteristics and demographics by study.

Study no.	2001	2004	3001	3002	3003	Total^†^
Number of subjects	22^a^	14	63	27	83	113
Median weight, kg (range)	76.5 (48.5–108.1)	63.2 (36.0–93.0)	70.0 (40.0–132.3)	22.0 (11.0–51.6)	64.0 (13.5–122.0)	63.1 (11.0–132.3)
Median age, years (range)	31 (15–58)	22 (13–46)	30 (12–61)	6 (1–10)	27 (2–63)	25 (1–61)
Median BMI, kg/m^2^ (range)	23.6 (16.0–35.3)	21.6 (16.4–31.4)	23.1 (17.1–63.1)	16.1 (12.8–26.9)	21.8 (13.1–39.8)	21.3 (12.8–63.1)
Median predose FIX activity, IU/dL (range)	0.7 (0.1–3.4)	1.6 (0.1–4.1)	1.9 (0.1–14.1)	1.6 (0.3–9.4)	16.9 (0.1–80.3)	1.3 (0.1–14.1)
Median baseline AST, IU/L (range)	23.1 (13.0–98.0)	23.5 (13.0–47.0)	23.0 (9.0–142.0)	30.5 (13.0–98.0)	28.0 (7.0–77.0)	25.0 (12.0–142.0)
Median baseline ALT, IU/L (range)	23.2 (13.0–117.0)	19.5 (12.0–74.0)	22.0 (9.0–102.0)	18.0 (8.0–154.0)	21.0 (8.0–107.0)	21.0 (8.0–154.0)
Median creatinine clearance, mL/min (range)	111.4 (85.4–218.3)	128.9 (101.7–149.4)	128.7 (67.5–300.5)	92.4 (56.7–159.8)	121.1 (58.1–229.3)	121.1 (56.7–300.5)
Hepatitis positivity, *N*						
Yes	15	2	3	0	22	23
No	7	12	60	27	61	90
Antidrug antibody, *N*						
Present	0	0	0	0	0	0
Absent	22	14	63	27	83	113
Region, *N*						
North America	0	0	6	0	3	69
Europe	20	3	36	22	49	73
Middle East	2	11	11	4	15	18
Asia Pacific	0	0	10	0	12	13
Australia	0	0	0	1	2	1
South Africa	0	0	0	0	2	2
Japanese, *N*						
Yes	0	0	10	0	9	10
No	20	14	53	27	74	113

Total represents the 113 unique patients; 76 of these participated in more than one study.

Where demographic values differ for patients across studies the first recorded value has been used in the summary.

BMI, body mass index; AST, aspartate transaminase; ALT, alanine transaminase; FIX, factor IX; IU, international unit.

^†^Total represents the 113 unique patients; some participated in more than one study.

a 22 subjects were enrolled, but only 20 were included in the PK analysis set due to missing data.

No development of anti-FIX inhibitors was observed in the analysis of pooled data from five clinical studies.

**Figure 1:**
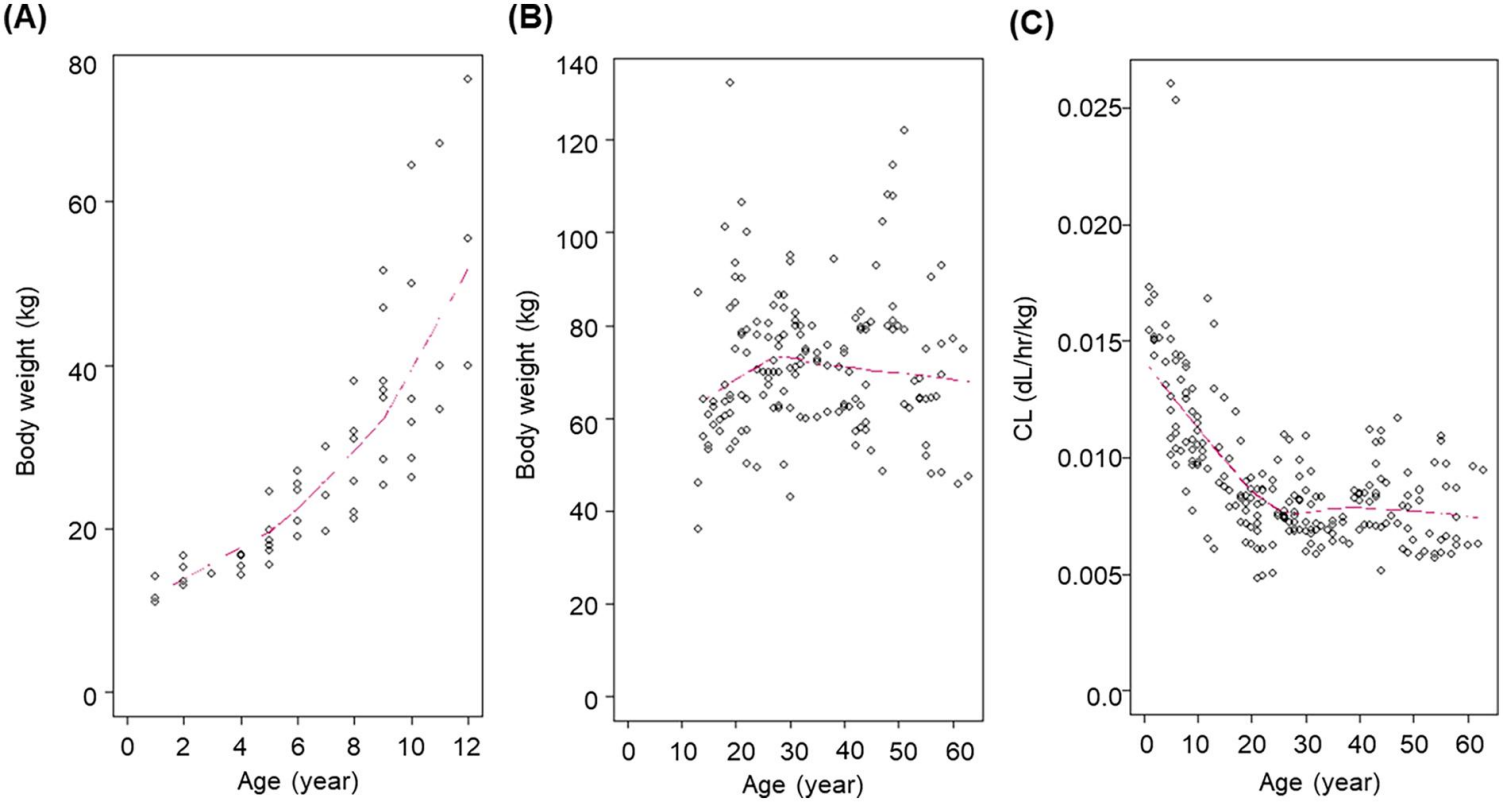
Relationship between body weight and age in patients aged ≤12 years **(A)** and >12 years **(B)**, and individual FIX clearance versus age **(C)** A similar plot for Vc was not included, as inter-individual variability in Vc was limited and no significant age-related trend was identified during model development. CL, clearance; FIX, factor IX.

Figures 1A,B shows the relationship between body weight and age. In patients aged ≤ 12 years, body weight strongly correlated with age; however, this correlation was not evident in patients aged > 12 years.

### Pharmacokinetic model parameters

FIX activity was well described by a 2-compartment population PK model with body weight on Vc, Vp and CL, and weight-adjusted dose on Vc. While weight-adjusted dose is mathematically dependent on body weight, it was retained as a significant covariate due to its independent contribution to improved model fit and predictive performance, without evidence of collinearity.

The parameters of the final PK model are shown in Table 2. The typical parameter estimates were 0.55 dL/h, 65.7 dL, 0.21 dL/h and 19.5 dL for CL, Vc, Q and Vp, respectively. Endogenous FIX activity (BASE) was estimated to be 0.99 IU/dL, with individual estimates being ≤ 2 IU/dL. The differences between the previous (11) and the present popPK models were minor. Inter-individual variability was applied on CL, V1 and BASE. Combined additive and proportional residual errors were estimated separately for Study 2001/2004 and Study 3001/3002/3003 (Table 2).

**Table 2:** Parameter estimates of final FIX activity population PK model.

Parameter, units	NONMEM estimates		
	Point estimate (95% CI)	% RSE	CV%
Structural model			
BASE, IU/dL	0.992 (0.657–1.33)	17.2	
CL, dL/h	0.550 (0.524–0.576)	2.44	
Vc, dL	65.7 (60.9–70.5)	3.76	
Q, dh/h	0.208 (0.0977–0.318)	27.1	
Vp, dL	19.5 (13.3–25.7)	16.1	
Weight-adjusted dose on Vc	0.281 (0.196–0.366)	15.4	
Body weight on Vc and Vp	0.771 (0.664–0.878)	7.11	
Body weight on CL	0.528 (0.438–0.618)	8.71	
Inter-individual variability^a^			
BASE, IU/dL	0.176 (0.0129–0.339)	47.3	42.0
CL, dL/h	0.0386 (0.0220–0.0552)	22.0	19.6
Vc, dL	0.0562 (0.0219–0.0905)	31.1	23.7
Residual variability^b^			
Study 2001/2004 (proportional)	0.181 (0.138–0.224)	12.2	18.1
Study 2001/2004, IU/dL (additive)	0.683 (0.171–1.19)	38.2	
Study 3001/3002/3003 (proportional)	0.371 (0.328–0.414)	5.93	37.1
Study 3001/3002/3003, IU/dL (additive)	1.20 (0.786–1.61)	17.6	

BASE, endogenous FIX activity; CI, confidence interval; CL, clearance; CV, coefficient of variation of proportional error; FIX, factor IX; IU, international unit; NONMEM, non-linear mixed-effect modeling software; Q, intercompartmental clearance; RSE, relative standard error; Vc, volume of central compartment; Vp, volume of peripheral compartment.

a Values shown represent the variance of random effect.

b Values shown represent either the proportional or additive components of the residual error model. The reference population weight for the pharmacokinetic parameters for Vc and Vp is a 70-kg patient. The reference weight-adjusted dose for Vc is 50 IU/kg.

Of note, and consistent with the clinical symptoms of the disease, the model-predicted endogenous FIX activity levels are lower than the observed pre-dose activity levels in the various studies contributing data, because of the aforementioned ability of the popPK model to account for previous FIX product dosing. The estimated residual variability for the proportional and additive components were higher for Studies 3001, 3002, and 3003 than for Studies 2001 and 2004, which is consistent with the higher observed variability for Studies 3001, 3002, and 3003.

Figure 1C shows the individual estimates of CL on a dL/h per kg weight basis over the range of ages of the patients included in the current dataset. The CL of FIX activity on a per kg weight basis was faster in patients <12 years compared to PTPs ≥12 years. The median value of the individual *post-hoc* estimates of CL on a per kg weight basis was 0.0155, 0.0114, 0.0103, 0.00836 and 0.00822 dL/h/kg for patients < 6 years, 6 to < 12 years, 12 to <18 years, ≥12 years and ≥ 18 years.

Although inter-individual variability was estimated for Vc, no statistically significant covariates, including age, were identified, and individual Vc estimates were not retained for further stratified analyses. Age was not retained in the final model after accounting for body weight, consistent with previous findings that weight-adjusted dosing eliminates most age-related variability in FIX exposure (19).

### FIX activity popPK model simulations

The popPK model was employed to estimate the likelihood of achieving target FIX activity levels, with simulations performed separately for four distinct age groups to assess the impact of age.

In adolescent (12–18 years) and adult (≥18 years) patients, a single IV infusion of rIX-FP at doses of 25, 40, 50, 75, and 100 IU/kg was predicted to maintain median FIX activity levels above 5% for 6.5/7.5, 9.5/11, 11.5/12.5, 15/16.5, and 18/20 days, respectively (Table 3). While for children aged 6 to <12 years, these durations were 5, 8, 9.5, 12.5, and 15 days, for those aged <6 years, the corresponding durations were 4, 6, 7.5, 10, and 9.5 days, respectively.

**Table 3:** Summary of the simulated durations for maintenance of exogenous FIX activity above 1%, 3%, and 5% after single administrations of rIX-FP by age.

Simulated rIX-FP dose FIX activity	Simulated duration (Days), Median (25th Percentile)				
	0 to <6 years	6 to <12 years	12 to <18 years	≧12 years	≧18 years
25 IU/kg					
>1%	11 (9.5)	13.5 (11.5)	16.5 (14)	18 (15)	18 (15.5)
>3%	6 (5)	8 (6.5)	9.5 (8)	10.5 (8.5)	10.5 (8.5)
>5%	4 (3.5)	5 (4.5)	6.5 (5.5)	7 (6)	7.5 (6)
40 IU/kg					
>1%	14 (12)	17 (14.5)	21 (17.5)	22.5 (19.5)	23 (19.5)
>3%	8.5 (7.5)	11 (9)	13 (11)	14.5 (12)	14.5 (12)
>5%	6 (5.5)	8 (6.5)	9.5 (8)	10.5 (9)	11 (9)
50 IU/kg					
>1%	15.5 (13)	19 (16)	23 (19.5)	25 (21.5)	25.5 (21.5)
>3%	10 (8.5)	12.5 (10.5)	15 (12.5)	16.5 (14)	16.5 (14)
>5%	7.5 (6.5)	9.5 (8)	11.5 (9.5)	12.5 (10.5)	12.5 (10.5)
75 IU/kg					
>1%	18 (15.5)	23 (19.5)	27.5 (23.5)	30 (25.5)	30 (25.5)
>3%	12.5 (10.5)	15.5 (13.5)	19 (16)	20.5 (17.5)	21 (17.5)
>5%	10 (8.5)	12.5 (10.5)	15 (13)	16.5 (14)	16.5 (14)
100 IU/kg					
>1%	21 (17.5)	25.5 (21.5)	31 (26)	33.5 (29)	34 (29)
>3%	14.5 (12.5)	18.5 (15.5)	22 (18.5)	24 (20.5)	24.5 (20.5)
>5%	12 (10)	15 (12.5)	18 (15)	19.5 (16.5)	20 (17)

The 25th percentile was used to reflect the lower end of the skewed distribution after single administration, in contrast to the 90% prediction intervals used for multiple doses in Table 4.

FIX, factor IX; IU, international unit; PK, pharmacokinetic; rIX-FP, recombinant fusion protein linking coagulation factor IX with albumin.

Multiple-dose/steady-state PK simulations explored the effect of reduced dosing frequency across all age groups (Table 4). In adolescents and adults, median trough exogenous FIX activity levels were maintained above 5 IU/dL throughout the dosing interval for 25, 40, and 50 IU/kg weekly regimens, 50 IU/kg every 10 days, 75 IU/kg every 14 days, and 100 IU/kg every 21 days (adults only). Similarly, children aged 6 to <12 years maintained levels above 5 IU/dL for 25, 40, and 50 IU/kg weekly regimens and 50 IU/kg every 10 days. However, in children aged <6 years, this was achieved only with 40 and 50 IU/kg weekly regimens.

**Table 4:** Summary of the simulated trough exogenous FIX activity after multiple administrations of rIX-FP by Age.

Parameter	Trough exogenous FIX activity (IU/dL), Median (90% PI)				
	0 to <6 years	6 to <12 years	12 to <18 years	≧12 years	≧18 years
25 IU/kg weekly	3.3 (1.4–6.6)	5.0 (2.2–9.9)	7.2 (3.3–13.4)	8.3 (3.9–15.6)	8.4 (4.0–15.8)
40 IU/kg weekly	5.8 (2.5–11.3)	8.8 (4.0–16.9)	12.5 (6.0–22.7)	14.5 (7.0–26.6)	14.7 (7.2–26.9)
50 IU/kg weekly	7.5 (3.4–14.6)	11.5 (5.3–21.8)	16.3 (7.9–29.3)	18.9 (9.2–34.1)	19.2 (9.5–34.5)
50 IU/kg every 10 days	3.6 (1.3–7.9)	5.9 (2.3–12.6)	8.5 (3.5–16.9)	10.1 (4.4–19.9)	10.3 (4.5–20.1)
50 IU/kg every 14 days	1.5 (0.4–4.0)	2.7 (0.8–6.8)	4.2 (1.5–9.4)	5.1 (1.9–11.3)	5.3 (2.0–11.5)
75 IU/kg every 14 days	2.6 (0.7–6.7)	4.6 (1.5–11.1)	7.1 (2.6–15.3)	8.6 (3.3–18.4)	8.8 (3.4–18.6)
75 IU/kg every 21 days	0.6 (0.1–2.5)	1.4 (0.3–4.6)	2.6 (0.7–6.9)	3.3 (1.0–8.5)	3.4 (1.0–8.6)
100 IU/kg every 21 days	1.0 (0.2–3.7)	2.1 (0.5–6.7)	3.8 (1.1–9.9)	4.8 (1.5–12.2)	5.0 (1.6–12.4)

FIX, factor IX; PI, prediction interval; PK, pharmacokinetic; rIX-FP, recombinant fusion protein linking coagulation factor IX with albumin.

The percentage of patients with simulated trough exogenous FIX activity above the cutoff was calculated across four age groups (Table 5). For the 50 IU/kg weekly regimen, nearly all patients in every age group were predicted to achieve a trough level of 5%. However, extending the dosing interval to 50 IU/kg every 10 days reduced this proportion to 25% in children aged <6 years. Further extending the interval to 50 IU/kg every 14 days resulted in 0% of children aged <6 years and only 13.3% of children aged 6 to <12 years achieving the target level. Even when increasing the dose to 75 IU/kg every 14 days, only 16.7% of children aged <6 years and 46.7% of children aged 6 to <12 years were predicted to reach the target. These findings indicate that extending the dosing interval significantly decreases the likelihood of achieving the target trough level in pediatric patients, particularly in children aged <6 years.

**Table 5:** Summary of % of patients with simulated trough exogenous FIX activity above 1%, 3%, or 5% following multiple doses of rIX-FP.

Simulated rIX-FP dose FIX activity	% of patients with simulated trough exogenous FIX activity above cutoff				
	0 to <6 years	6 to <12 years	12 to <18 years	≧12 years	≧18 years
25 IU/kg every 7 days					
>1%	100	100	100	100	100
>3%	58.3	86.7	100	98.8	98.7
>5%	16.7	53.3	77.8	87.2	88.3
40 IU/kg every 7 days					
>1%	100	100	100	100	100
>3%	91.7	100	100	100	100
>5%	58.3	86.7	100	98.8	98.7
50 IU/kg every 7 days					
>1%	100	100	100	100	100
>3%	100	100	100	100	100
>5%	83.3	100	100	100	100
50 IU/kg every 10 days					
>1%	100	100	100	100	100
>3%	66.7	86.7	100	98.8	100
>5%	25.0	60.0	88.9	91.9	93.5
50 IU/kg every 14 days					
>1%	66.7	93.3	100	100	100
>3%	8.3	40.0	77.8	82.6	84.4
>5%	0.0	13.3	33.3	52.3	53.2
75 IU/kg every 14 days					
>1%	91.7	100	100	100	100
>3%	41.7	73.3	88.9	96.5	97.4
>5%	16.7	46.7	77.8	83.7	85.7
75 IU/kg every 21 days					
>1%	33.3	66.7	88.9	95.3	96.1
>3%	0.0	13.3	44.4	55.8	57.1
>5%	0.0	0.0	11.1	24.4	26.0
100 IU/kg every 21 days					
>1%	50.0	80.0	100	98.8	98.7
>3%	8.3	33.3	66.7	76.7	77.9
>5%	0.0	13.3	33.3	47.7	49.4

FIX, factor IX; IU, international unit; rIX-FP, recombinant fusion protein linking coagulation factor IX with albumin.

### Japanese patient population

Using the same global patient popPK dataset and the same nonlinear mixed effects model as described above, covariate testing of Japanese ethnicity (Japanese vs. non-Japanese) was performed using the updated model-based PK sub-analysis. Consistent with global patient population, the PK of FIX activity after administration of rIX-FP was characterized well by a 2-compartmental model, with body weight and weight-adjusted dose being the only 2 significant covariates. Japanese ethnicity was not a significant model covariate.

A summary comparison of the PK parameters and their variability between Japanese and non-Japanese patients is presented in Table 6. CL values were 0.00823 dL/h/kg in Japanese patients and 0.00957 dL/h/kg in non-Japanese patients. These results suggest that the PK profile of rIX-FP does not differ significantly between Japanese and non-Japanese patients.

**Table 6:** Comparison of body weight-adjusted PK parameters between Japanese and non-Japanese patients.

Parameter, units	Japanese patients *N* = 10	Non-Japanese patients *N* = 101
CL, dL/h/kg		
Mead (SD)	0.00823 (0.00162)	0.00957 (0.00337)
Median (min, max)	0.00845 (0.00594, 0.0109)	0.00857 (0.00480, 0.0261)
Vc, d/kg		
Mead (SD)	1.07 (0.331)	1.01 (0.310)
Median (min, max)	0.969 (0.862, 1.97)	0.940 (0.489, 2.49)
Vp, d/kg		
Mead (SD)	1.36 (0.339)	1.31 (0.336)
Median (min, max)	1.26 (1.15, 2.28)	1.24 (0.759, 2.80)

CL, clearance; max, maximum; min, minimum; PK, pharmacokinetic; Vc, central volume of distribution; Vp, peripheral volume of distribution.

The PK simulation profile at steady-state for 100 IU/kg rIX-FP administrated every 21 days showed comparable and overlapping PK FIX activity profiles for Japanese and non-Japanese patients. The median trough FIX activity at 3 weeks for Japanese and non-Japanese patients was 4.09 and 3.98 IU/dL, respectively (Table 7).

**Table 7:** Summary of the simulated PK parameters and variability after administration of 100 IU/kg rIX-FP every 21 days.

Parameter, units	Median (90% PI)	
	Japanese patients *N* = 10	Non-Japanese patients *N* = 101
C_trough_, IU/dL	4.09 (1.19, 10.3)	3.98 (0.603, 11.5)
C_max_, IU/dL	87.9 (62.5, 126)	87.7 (55.7, 130)
AUC_ss_, IU × h/dL	11,528 (7,373, 17,208)	11,576 (5,492, 18,824)

AUC_ss_, AUC at steady-state; C_max_, maximum concentration; C_trough_, concentration at trough; IU, international unit; PI, prediction interval; PK, pharmacokinetic; rIX-FP, recombinant fusion protein linking coagulation factor IX with albumin.

## Discussion

The present study provides clinicians with PK-based insights to better understand rIX-FP therapy and determine clinically relevant optimal dosing strategies. This model was used to support the regulatory approval of an extended 3-week dosing regimen for rIX-FP in patients aged ≥12 years, based on the predicted ability to maintain target FIX activity trough levels at steady state. The findings suggest that rIX-FP can consistently reach adequate target FIX activity trough levels while reducing treatment burden and drug consumption. However, the model also indicates that certain targets may not be achievable or may only be reached in a small subset of patients with HB.

This model for rIX-FP, developed using pooled data from five clinical PK studies that included pediatric, adolescent, and adult patients, demonstrated reliable parameter estimation with high precision, aligning with observed clinical trial data. Additionally, the predicted steady-state FIX activity levels across various doses and dosing intervals suggest that extended dosing intervals of 7 days up to 3 weeks are feasible while maintaining high FIX trough levels in well-controlled patients. As a result, the PCA to update the rIX-FP IV product information with this regimen has been approved in Japan and EU. However, the 3-week interval is authorized for patients aged ≥12 years in Japan and for those aged ≥18 years in the EU. This regulatory approval was primarily supported by the clinical efficacy and safety results from Study CSL654_3003 (13, 14), with the present PopPK analysis providing supplementary pharmacokinetic evidence for the feasibility of extended dosing intervals.

Potential limitation of the model is the presence of residual FIX activity from prior FIX products in some subjects at baseline. To address this, the model incorporated an exponentially declining residual component (FPP) and constrained the endogenous FIX activity to ≤2 IU/dL, consistent with inclusion criteria. This approach allowed for separation of endogenous and residual exogenous FIX activity, minimizing bias in parameter estimation and trough level predictions.

Maintaining trough FIX activity levels above 5% is a key clinical target for HB prophylaxis, as symptoms are typically categorized as “mild” under these conditions (1, 2, 17). For patients aged ≥12 years, a single 100 IU/kg dose of rIX-FP maintained FIX activity at 5% for approximately 20 h, and repeated 3-week dosing achieved a median trough FIX activity of 4.8%, demonstrating its ability to sustain adequate trough levels with lower weekly dose and administration frequency compared to other FIX replacement therapies (12). Although this median trough level was slightly below the nominal target of 5 IU/dL, it is very close to the range typically associated with mild hemophilia. Individual treatment decisions should take into account a patient's endogenous FIX activity and clinical characteristics, and in some cases, more frequent dosing may be warranted to maintain desired protection.

It should be noted, however, that a median steady-state trough level of approximately 5 IU/dL implies that roughly half of the population may fall below this threshold. The clinical relevance of a 5% trough level may also vary across extended half-life FIX products due to differences in PK–PD relationships. For example, Koopman et al. (20) demonstrated that even with similar target trough levels, bleeding outcomes may differ, indicating that trough activity alone does not fully capture clinical efficacy. Moreover, while trough levels serve as a convenient proxy for protection, other PK metrics such as peak FIX activity and time above a certain threshold (e.g., for physical activity) may also be clinically important. In this context, it is reassuring that the 100 IU/kg every-3-weeks regimen was prospectively evaluated in Study CSL654_3003, which demonstrated clinical safety and no thrombotic events attributable to peak levels. Nonetheless, we acknowledge that further evaluation of time–concentration profiles and individual variability may support a more nuanced interpretation of prophylactic efficacy and patient-specific needs.

However, for pediatric patients aged 0 to <12 years, rIX-FP 100 IU/kg every 3 weeks resulted in median trough FIX activity of only 1.0% and 2.1%, respectively, necessitating weekly dosing at 50 IU/kg to maintain 5%. This highlights the limitation of the 3-week regimen in younger patients, which can be attributed to their higher clearance per kg body weight. The median value of CL on a per kg weight basis was notably faster in pediatric patients, at 1.55 and 1.14 mL/h/kg for the 0 to <6 and 6 to <12 age groups, respectively, compared to 0.836 mL/h/kg in those ≥12 years. By comparison, other EHL products, such as rFIX-Fc and N9-GP, require dosing every 10–14 days (5, 21). Similar findings have been reported for rFIX-Fc, highlighting the faster clearance in pediatric patients (22).

Consistent with prior popPK modeling (11), weight-based allometric scaling of clearance was identified as a significant determinant of clearance, with an estimated allometric exponent of 0.53 (95% CI: 0.44–0.62), which is lower than the theoretical value of 0.75. This finding suggests that pediatric patients exhibit higher clearance per kg body weight, primarily due to their lower body weight and increased metabolic or elimination rates. Generally, both per-kg FIX clearance (CL/kg) and per-kg central volume of distribution (Vc/kg) are larger in children than in adults, with clearance and volume of distribution decreasing as age increases (23). A similar pattern has been reported for recombinant factor VIII in hemophilia A patients (24). In this study, no significant effect of age was observed, likely due to the strong correlation between age and body weight in pediatric patients younger than 12 years. While age itself may not be a determining factor, the impact of body weight on clearance underscores the need for individualized dosing in younger patients.

Several continuous covariates, including BMI, AST, ALT, and creatinine clearance, were evaluated, but none demonstrated a significant association with CL. In addition, an assessment of the impact of Japanese ethnicity showed no significant effect, resulting in its exclusion from the model. Importantly, simulations for the 21-day prophylactic regimen with 100 IU/kg rIX-FP demonstrated comparable pharmacokinetic profiles in Japanese patients and the broader global population.

In addition, body weight–adjusted dose was found to be a significant covariate for central volume of distribution (Vc), with higher doses associated with increased Vc. This finding may reflect a potential dose-dependent distribution behavior or a nonlinear pharmacokinetic process, such as saturation of binding sites or altered tissue distribution at higher concentrations. While the current model does not explicitly include nonlinear kinetics, similar covariate relationships have been reported for other extended half-life FIX products. In previous population PK analyses of rFIXFc, for example, body weight was identified as a significant covariate for both clearance and Vc (22). Although similar analyses of N9-GP have also been conducted (25), explicit relationships between body weight and Vc variability have not been clearly documented to date. These findings support the biological plausibility of body size–related changes in drug distribution, which may manifest as apparent dose-dependent Vc variation in clinical settings. Further investigation would be required to elucidate whether this reflects true nonlinear distribution kinetics or a covariate-driven scaling effect based on physiological characteristics such as body mass.

Across all age groups, EHL rFIX products exhibit markedly improved PK profiles compared to standard SHL FIX products. For rIX-FP, clearance in children aged 0 to <6 years is nearly one-sixth of that seen with standard SHL FIX products (10). While higher clearance in younger patients is often attributed to physiological factors such as larger plasma volume per body weight or differences in protein binding, these conventional explanations do not fully account for the pronounced clearance differences observed with EHL rFIX products like rIX-FP. One possible explanation involves the neonatal Fc receptor (FcRn). rIX-FP, as an albumin-fusion protein, interacts with FcRn, which prevents lysosomal degradation and allows albumin to be recycled similarly to IgG, thereby extending its half-life (26, 27). FcRn plays a critical role in neonatal immune protection by transporting maternal IgG across the placenta or neonatal gut. In early infancy, IgG transport may be prioritized over albumin recycling, potentially reducing the retention of albumin-fused molecules such as rIX-FP. As the neonate's immune system matures and endogenous IgG production increases, FcRn binding dynamics and recycling efficiency may shift with reduced priority for IgG over albumin, contributing to observed age-related differences in rIX-FP clearance with the lower allometric exponent than the theoretical value (27). Given the relatively high incidence of intracranial hemorrhage in hemophilia B, particularly among neonates (28), proactive management through shared decision-making involving clinicians, patients, and caregivers is essential.

## Conclusions

This popPK model provides valuable insights for optimizing FIX replacement therapy in HB, ensuring that target steady-state FIX trough levels can be achieved while minimizing dosing frequency. Our findings support rIX-FP as one of the extended half-life FIX products with a 3-week dosing option, which may offer patients and clinicians increased flexibility in treatment planning to reduce administration frequency, depending on individual needs. While pediatric patients require more frequent dosing due to their higher clearance rates, this model serves as an essential tool for guiding shared decision-making between clinicians, caregivers, and patients, allowing for individualized treatment approaches that align with patient needs and preferences. Ultimately, the application of this model in clinical practice may enhance treatment outcomes, improve adherence, and increase overall patient satisfaction.

## Data Availability

Publicly available datasets were analyzed in this study. This data can be found here: the de-identified datasets analyzed in this study are not publicly available because they were obtained from the sponsor (CSL Behring) under a data use agreement. Requests for access to these data should be directed to the sponsor. Summary-level results and analysis code supporting this article are available from the corresponding author upon reasonable request.

## Supplementary material

Supplementary Figure S1 Goodness of fit plots of the population pharmacokinetic model. The black solid lines represent the unity line and the red dashed lines the local regression smoothing line. FIX, factor IX; hr, hour.

Supplementary Figure S2 Visual predictive check for the final FIX activity population PK model after rFIX-FP administration. Open circle: prediction-normalized observed concentrations; solid line: median of observed concentrations; dashed lines: 5th and 95th percentile of prediction-normalized observed concentrations. Red shaded region: 90% prediction interval for median of prediction-normalized predicted concentrations; blue shaded regions: 90% prediction intervals for the 5th and 95th percentiles of prediction-normalized predicted concentrations. The x-axis represents time after the last dose.
