# Supplementary figures and images for: Optimization of hemophilia B treatment via population PK modeling of rIX-FP, including a 3-week regimen
Source: Front Pediatr. 2025 Dec 15;13:1710546. doi: 10.3389/fped.2025.1710546 (PMC12745456; doi:10.3389/fped.2025.1710546)

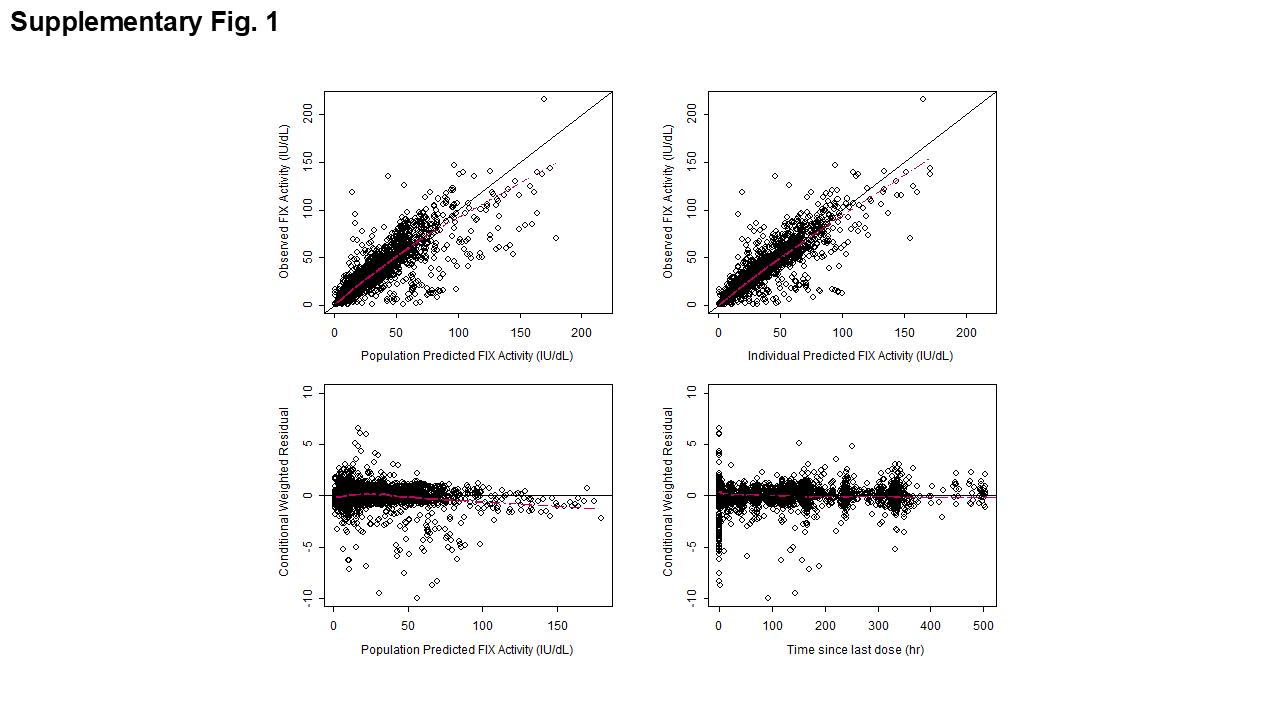

Supplement: Supplementary Figure S1 — Goodness of fit plots of the population pharmacokinetic model. The black solid lines represent the unity line and the red dashed lines the local regression smoothing line. FIX, factor IX; hr, hour. [file Image1.tif]

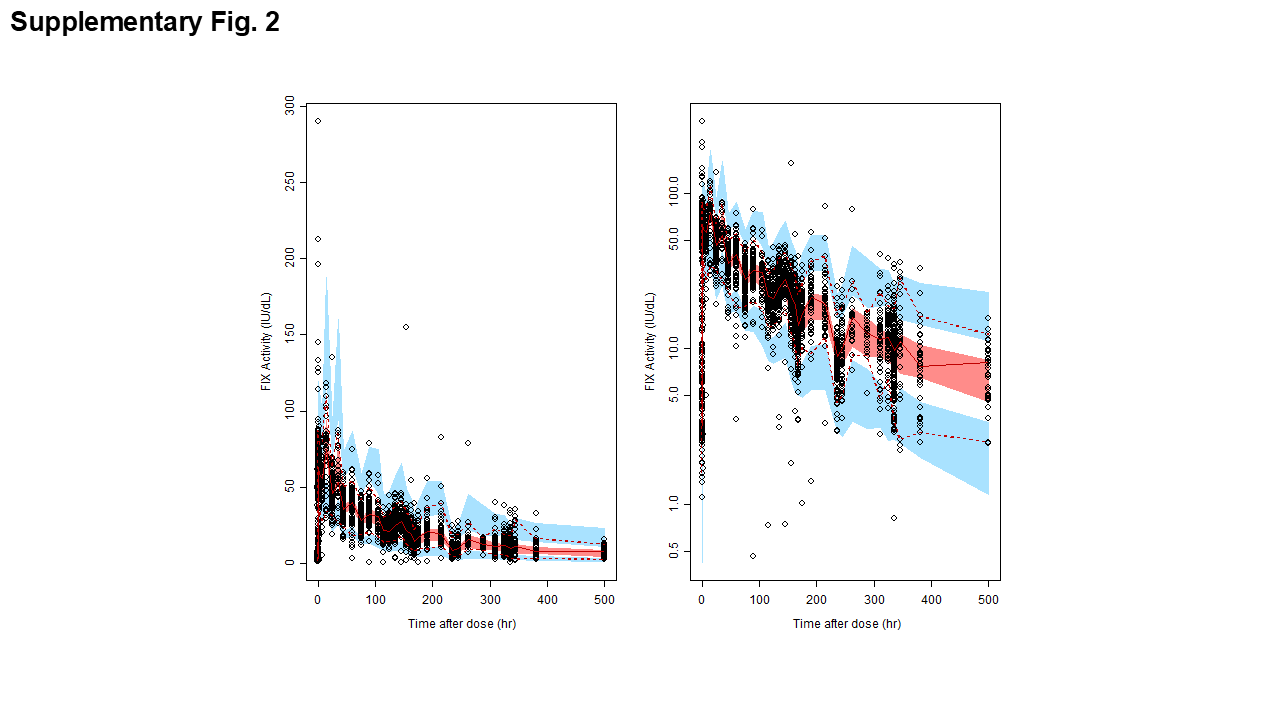

Supplement: Supplementary Figure S2 — Visual predictive check for the final FIX activity population PK model after rFIX-FP administration. Open circle: prediction-normalized observed concentrations; solid line: median of observed concentrations; dashed lines: 5th and 95th percentile of prediction-normalized observed concentrations. Red shaded region: 90% prediction interval for median of prediction-normalized predicted concentrations; blue shaded regions: 90% prediction intervals for the 5th and 95th percentiles of prediction-normalized predicted concentrations. The x-axis represents time after the last dose. [file Image2.tif]
