# Supplementary material for: Optimization of hemophilia B treatment via population PK modeling of rIX-FP, including a 3-week regimen
Source: Front Pediatr. 2025 Dec 15;13:1710546. doi: 10.3389/fped.2025.1710546 (PMC12745456; doi:10.3389/fped.2025.1710546)
Supplement: Supplementary file 3 [file Table1.docx]

**Supplementary Table S1** Overview of study patients and dosing

| **Study No.** | **2001** | **2004** | **3001** | **3002** | **3003** |
| --- | --- | --- | --- | --- | --- |
| Phase | Phase 1 | Phase 1/2 | Phase 2/3 | Phase 3 | Phase 3b |
| Type of study | Safety, PK | Safety, Efficacy, PK | Safety, Efficacy, PK | Safety, Efficacy, PK | Safety, Efficacy, PK |
| Number of subjects | 22 | 14 | 63 | 27 | 83 |
| Age | 15-58 years | 13-46 years | 12-61 years | < 12 years | 2-63 years |
| Previously treated | Yes | Yes | Yes | Yes | Yes |
| IV Dose | 25 IU/kg rIX-FP (n = 8)  50 IU/kg rIX-FP (n = 14)  75 IU/kg rIX-FP (n = 8) | 25 IU/kg rIX-FP during single dose PK assessment;  Weekly 15-35, up to 75 IU/kg after single dose PK assessment | 25 or 50 or 75 IU/kg rIX-FP (except the subjects from 654-2004);  ~15 subjects repeated the 50 IU/kg PK at 6 months | 50 IU/kg rIX-FP during single dose PK assessment;  PK of the previous 50 IU/kg FIX product in a sub-set of subjects | 30 to 50 IU/kg rIX-FP every 14 days to 100 IU/kg every 21 days  Arm 1: Subjects who completed previous 654 studies; dosing every 7, 10 or 14 days  Arm 2: Subjects enrolled on 654_3001; dosing every 7 or 14 days  Arm 3: Subjects not completed a previous 654 study; dosing every 7 days |

FIX, factor X; IU, international unit; IV, intravenous; PK, pharmacokinetic; rIX-FP, recombinant fusion protein linking coagulation factor IX with albumin
